# Improved Tolerance of *Artemisia ordosica* to Drought Stress via Dark Septate Endophyte (DSE) Symbiosis

**DOI:** 10.3390/jof8070730

**Published:** 2022-07-13

**Authors:** Xia Li, Xue Zhang, Minghui Xu, Qiannan Ye, Huili Gao, Xueli He

**Affiliations:** 1 School of Life Sciences, Hebei University, Baoding 071002, China; zhaolili@hbu.edu.cn (X.Z.); mhx0631@126.com (M.X.); yqn5100@163.com (Q.Y.); ylzhy@hebau.edu.cn (H.G.); 2 Key Laboratory of Microbial Diversity Research and Application of Hebei Province, School of Life Sciences, Hebei University, Baoding 071002, China

**Keywords:** dark septate endophyte, desert, *Artemisia ordosica*, species diversity, drought stress

## Abstract

Dark septate endophytes (DSEs) usually colonize plant roots, especially in stress environments. However, their relationship with plants ranges from beneficial to harmful and has remained largely uncharacterized. In the present study, 14 DSE species grouped into 11 genera were isolated from the roots of a desert plant, *Artemisia ordosica*, which is widely distributed in northwest China. Three dominant DSE species—*Paraphoma chrysanthemicola* (Pc), *Alternaria chartarum* (Ac), and *Acrocalymma vagum* (Av)—were selected and tested for their resistance to drought in vitro. Furthermore, we characterized the responses of *A. ordosica* under drought conditions in relation to the presence of these DSEs following inoculation. The results showed that all three strains grew well under in vitro drought stress, and the biomass of Ac and Av was significantly higher than that of the unstressed control. The effects of DSE inoculation on the growth of *A. ordosica* under drought stress varied according to the different DSE species but were generally beneficial. Under drought stress, Av and Pc promoted plant growth, antioxidant enzyme activity, and root development of the hosts. The Ac strain conferred obvious positive effects on the antioxidant enzyme activity of the hosts. In general, Av and Pc demonstrated better application potential for improving the drought resistance of *A. ordosica*.

## 1. Introduction

As one of the major ecological problems facing the world, desertification has great impact on the structure and function of soil [1]. This phenomenon results in a loss of soil nutrients and a decrease in vegetation productivity, which seriously reduces the living space of organisms and restricts the sustainable development of the local environment and social economy [2,3,4,5]. In addition, desert ecosystems may be more vulnerable to climate change compared to other ecosystems [6,7,8]. In China, deserts cover approximately one-fifth of the country’s surface, and the conditions are particularly harsh in the arid regions of northwest China due to their sparse rainfall, arid climate, and strong wind and sand activity [9,10]. For a long time, land desertification has not only affected vegetation growth and biodiversity in this region but also seriously threatened the healthy development of agriculture, husbandry production, and food security [10,11]. Therefore, the restoration of vegetation and soil function in this area is essential for the sustainable development of the desert ecosystem.

During the long process of their evolution, desert plants have not only developed the ability to regulate their growth characteristics but also to respond to climate change and environmental stress by establishing symbioses with fungi [12,13]. Among them, root–symbiotic fungi interactions can promote nutrient absorption and stress resistance in hosts and regulate the relationship between plants and other soil microorganisms [14]. Based on these effects, mycorrhizal symbionts can influence plant root growth and regulate plant–soil interactions, thus affecting the processes and nutrient cycling of the ecosystem in which they are located [15]. Dark septate endophytes (DSEs), as a kind of endophyte in plant roots, act as a bridge connecting material transport between aboveground and underground ecosystems. Studies have shown that these fungi can enhance host adaptability by improving plant tolerance to multiple biological and abiotic stresses, resulting in increased biomass accumulation and reduced water consumption, and thus have a profound impact on plant growth and community construction [16,17,18].

DSEs form an ambiguous group and may be represented by several orders within ascomycetous fungi that colonize cells or intercellular spaces of healthy plant root tissues [16]. It has been reported that DSEs have a wide range of host and ecological distributions, showing an especially high colonization rate in the roots of plants under extreme climatic conditions (drought, cold, salt, etc.) or in contaminated environments [16,17,18,19]. DSEs cannot be identified based on colonization morphology alone; therefore, investigations using laboratory and molecular techniques are needed. In recent years, the species diversity and ecological functions of DSEs in arid or desert habitats have been studied [17,20,21,22,23]. Rayment et al. [24] studied nine plants distributed along the east coast of Australia and found that DSEs were widely distributed in their roots and preferred to grow in arid environments. Barrow [25] investigated *Bouteloua* sp., a plant in the semiarid grasslands of the United States, and found that the roots of these plants all showed DSE colonization. It was speculated that the DSE mycelia extending from the roots of the plants could help symbiotic plants maintain water and nutrient transport in arid environments and improve their drought resistance. Moreover, the symbiotic association between licorice plants and DSE fungi was shown to help licorice plants recover from drought stress [26]. These reports show that DSEs are widely distributed in areas of water stress, and colonization host plant roots by DSEs can help to increase tolerance of host plants to drought stress under in vitro culture conditions.

Despite the harsh conditions, desert ecosystems often comprise multizonal vegetation, and species that have typical characteristic of adaptation to drought, which is the main property determining desert productivity [9,27]. Among these species, *Artemisia ordosica* is one of the most dominant subshrubs and is distributed widely in the sandy areas of northwest China, such as Inner Mongolia, Ningxia, Shaanxi, Gansu, and other provinces [28,29,30]. *Artemisia ordosica* can adapt well to water stress conditions and has developed the typical morphological and physiological characteristics of adaptation to drought and sand burial [29,31]. For example, it has a taproot system, consisting of one large vertical root that produces many small lateral roots in a well-developed system. In addition, *A. ordosica* can not only reproduce by seed but can also produce a large number of adventitious roots from its branches and grow rapidly. Therefore, it has become the winner of the competition for plant survival in sandy environments and plays an important role in preventing wind erosion and fixing sand in northwest China [28,32,33]. Previous studies on these plants have mostly focused on their physiological characteristics, drought resistance, and ecological processes, while relatively few studies have focused on their symbiotic DSE fungi.

Microorganisms also play important roles in determining soil structure and aggregate formation as well as in functions related to plant health and growth [15,34,35]. Thus, the interaction between plants and microorganisms is an important part of desert ecosystems. Revealing the interaction between plants, microorganisms, and the environment can profoundly increase our understanding of the evolutionary process of desert ecosystems, which is of great significance for the restoration of desert vegetation [32]. The survival strategies and maintenance mechanisms of these organisms have become a hot topic in research. In addition, although DSEs may improve the growth and nutrition of the host plant, the nature of DSE symbiosis, namely whether it is beneficial or harmful for the host plant, has remained largely unclear [36]. Therefore, studies on the distribution and ecological function of DSEs in extreme environments would improve our understanding of the biology of DSE symbiosis. We assume that in a highly heterogeneous desert environment, the growth of *A. ordosica* not only strongly affects plant species diversity but also has a profound impact on the distribution and activities of DSE fungi that form a symbiotic relationship with the plant roots.

The deserts of northwest China are typically habitats of extreme stress and thus constitute an ideal area to study the effects of drought stress on plant–fungi symbiosis [23,37,38,39]. In this study, the DSE colonization status and species composition of *A. ordosica* in arid deserts were investigated in a field investigation, in which DSE strains were isolated. Subsequently, the influence of the isolated DSE strains on the growth and drought resistance of the host plant were assessed in a greenhouse assay. We hypothesized that DSE strains isolated from deserts would be drought-tolerant and have beneficial effects on host growth under drought stress conditions.

## 2. Materials and Methods

### 2.1. Study Sites

The root samples of *A. ordosica* were collected from three sites in northwest China: Shapotou (37.46° N, 105° E) in Ningxia, Alxa (38.98° N, 105.85° E), and Dengkou (40.40° N, 107.01° E) in Inner Mongolia. These areas have a typical temperate continental climate. The altitude ranges from 1000 to 1700 m, the average annual temperature is 7–10 °C, and the average annual rainfall is 186.6, 206.2, and 144.5 mm, respectively.

### 2.2. Soil and Root Sampling

Three small-scale plots were selected for each site in July 2018, in which five healthy *A. ordosica* plants were randomly selected. For each plant, four 7 cm soil column were collected in different directions at a depth of 30 cm. The soil samples were mixed and sieved (<2 mm mesh) to extract fine roots. The fine roots and soil samples were then placed into self-sealing bags and transported to the laboratory in an insulated container. All root samples were stored in refrigerator at 4 °C, and about 30 root segments/plant were used for DSE isolation.

### 2.3. Isolation and Identification of DSE Strains

Fresh root samples were washed with sterilized water, sterilized with 75% ethanol for 5 min, and then with 5% sodium hypochlorite for 2 min before being rinsed three times in sterilized water. The root samples were dried on sterilized filter paper and cut into 1 cm long segments, which were placed into potato dextrose agar (PDA) culture medium with antibiotics (containing 50 mg/L of ampicillin and streptomycin sulfate) in Petri dishes. Approximately five root segments were placed in each dish. These dishes were then incubated at 27 °C in the dark and observed daily. Colonies with dark mycelia were transferred to a new PDA plate [38].

The DSE strains were identified by morphological and molecular methods [40]. First, the morphological and microscopic characteristics of these isolates were determined for preliminary identification. Then, we conducted the molecular identification process. Approximately 50 mg of the mycelium from each colony was scraped and then placed into a 1.5 mL centrifuge tube and thoroughly ground. DNA was then extracted using a fungal genomic DNA extraction kit (Solarbio, Beijing, China). The PCR reaction system was 20 μL, containing 2× Taq PCR Master Mix (10 μL), template (3.5 μL), primers (0.5 μL each; 10 μmol/L stocks of ITS4 (5′-TCCTCCGCTTATTGATATGC-3′) and ITS5 (5′-GGAAGTAAAAGTCGTAACAAGG-3′)), and ddH_2_O (5.5 μL). The PCR process was performed in a Life ECO™ thermocycler (BIOER, China) (initial denaturation at 94 °C for 5 min, then 35 cycles of denaturation at 94 °C for 1 min, primer annealing at 55 °C for 1 min, extension at 72 °C for 1 min, and then a final extension at 72 °C for 10 min). Finally, the PCR products were purified and sequenced. The obtained DNA sequence was submitted to and used in BLAST searching of the GenBank database, and a phylogenetic tree was constructed using MEGA 6 software based on the ML algorithm [41]. The DNA sequences were stored in GenBank according to the following accession numbers: MK753038–MK753043, MK753045, MK753047, MK753049, MK753052–MK753056, MN592645, MN592646, MN592650, MN592652, MN592653, and MN592658. The DSE isolation frequency (IF) calculation refers to the isolate number of each fungus divided by the total number of fungal isolates.

### 2.4. DSE Colonization and Diversity Analysis

The DSE colonization of *A. ordosica* was detected according to the method of Phillips and Hayman [42]. Thirty fresh root segments (cut into 1 cm pieces) from each sample were randomly selected, incubated with 10% KOH at 100 °C for 1 h, and stained with 0.5% acid fuchsin at 90 °C for 5 min before being decolorized with lactic acid–glycerin for more than 12 h. The colonization structure and infection status of the DSEs were observed under an optical microscope, and the colonization rate and colonization intensity of the DSEs were calculated according to the following formula [22]:

Colonization rate (%) = number of infected root segments/total number of evaluated root segments × 100%

The DSE diversity was assessed using indices of Shannon–Weaver index (H). Dominance was calculated with Simpson’s dominance (D) measures. Evenness index (J) was used to determine of uniformity of the isolated DSE fungi. The formulae were as follows [43,44,45]:

H = − ∑(*Pi*)(In*Pi*)

D = 1 − ∑(*Pi*)^2^

where the ratio ‘*Pi*’ is the frequency of colonization of the taxon in the sample.

J = H/In(*S*)

where ‘*S*’ is the total number of DSE isolated.

### 2.5. Soil Physicochemical Properties

Soil organic matter was estimated by the combustion method, with samples being heated in a muffle furnace (TMF-4-10T, Shanghai Gemtop Scientific Instrument Corporation, Shanghai, China) at 550 °C for 4 h [46]. The soil available phosphorus (AP) was measured using the sodium bicarbonate molybdenum antimony anti-colorimetric method [47]. The soil available nitrogen (AN) was determined using the alkaline hydrolysis–diffusion method [48]. The soil pH was measured using a portable high-precision in situ pH meter (STEP pH 3000). The soil moisture was determined with a soil humidity recorder (L99-TWS-2, China). The soil acid phosphatase (ACP) and alkaline phosphatase (ALP) activities were determined using the Tarafdar and Marschner methods [49]. The soil urease (U) activity was measured according to the method described by Hoffmann and Teicher [50].

### 2.6. Drought Stress of the DSE Strains in Vitro

Three dominant DSE strains isolated from *A. ordosica* with the highest IFs were selected for this test (*A. chartarum*, *P. chrysanthemicola*, and *A. vagum*). These fungi were stored in the Laboratory of Plant Ecology, Hebei University, China. The capacity of the DSE isolates to grow under drought stress was tested in a liquid culture. Prior to the stress procedure, the three fungal strains were individually grown in potato dextrose agar (PDA) culture medium at 27 °C for two weeks. The experiment was conducted under sterile conditions using a modified Melin Norkrans (MMN) medium (glucose, 15.0 g; MgSO_4_·7H_2_O, 0.15 g; citric acid, 0.2 g; (NH_4_)_2_HPO_4_, 0.25 g; KH_2_PO_4_, 0.5 g; CaCl_2_, 0.05 g; NaCl, 0.025 g; VB_1_, 100 μg; FeCl_3_ (1%), 1.2 mL; distilled water, 1000 mL; pH 5.5). Polyethylene glycol 6000 (PEG-6000) was added to simulate drought stress, and the permeability gradient of PEG was 0 and 1.34 MPa [38,51]. For each strain, one fungal disk (5 mm in diameter) was cut from the edge of the colony and transferred to a sterilized 250 mL conical glass flask containing 150 mL of sterilized MMN medium. The cultures were incubated at 27 °C under constant shaking at 120 rpm for 15 days. Each treatment was repeated three times.

The mycelia were collected by filtering the fungal solution. The fresh mycelia were then randomly divided into two parts. One part was used to determine the superoxide dismutase (SOD) activity, malondialdehyde (MDA) content, soluble protein, and melanin content. The SOD activity was analyzed by the NBT photoreduction method [52]. MDA content was determined according to the thiobarbituric acid (TBA) method [53]. Soluble protein content was measured via Coomassie brilliant blue G-250 colorimetry [54]. Melanin was extracted from mycelia following the method described by Zhan et al. [55,56]. The other part was dried to a constant weight at 75 °C to measure the water content of the mycelia. The sum of the dry weights of these two parts was then taken as the total biomass of the fungi.

### 2.7. Inoculation Assay

#### 2.7.1. DSE Inoculation and Plant Growth Conditions

The seeds of *A. ordosica* were collected from the Desert Forestry Experimental Center, Chinese Academy of Forestry, Dengkou, and stored at 4 °C. Healthy seeds of the same size were selected, sterilized with 75% ethanol for 1 min, washed with sterile water several times, and then germinated in a sterilized culture dish with double-layer filter paper for three days. Following germination, the seedlings were planted in culture pots (bottom diameter, 5.5 cm; top diameter, 9.5 cm; height, 11.5 cm), filled with 400 g of 2 mm sieved autoclaved soil. The experimental soil contained 4.82 mg/g of organic carbon, 60.67 μg/g of alkali nitrogen, and 9.7 μg/g of available phosphorus (pH 6.73). Two seedlings were planted in each pot.

Two factors were examined in this experiment, namely, DSE inoculation and drought treatment, with four variables for each condition. The DSE inoculation treatments comprised a non-DSE inoculated control (CK) and *A. chartarum* (Ac), *P. chrysanthemicola* (Pc), and *A. vagum* (Av) inoculation. For the inoculation treatments, 5 mm fungal mycelia discs excised from the edge of the colonies actively growing DSE in potato dextrose agar (PDA) culture medium were placed 1 cm below the roots of the plants (two discs for each plant) in the culture pots. For the CK treatment, discs of the PDA culture medium without fungi were added into the culture pots. All of the inoculation processes were performed in a sterile environment. The culture pots were placed in an illumination incubator with a light cycle of 14 h light/10 h dark, a temperature of 27 °C light/22 °C dark, and 50% mean relative humidity. One month after sowing, the seedlings were treated with drought stress. The drought stress treatments included drought (W−) or well-watered (W+) treatment, corresponding to 30% and 70% of the field water capacity, respectively. Water loss was supplemented daily with sterile distilled water to maintain the desired field capacity by regular weighing. The plant growth and physiological parameters, as well as root colonization, were measured 90 days after sowing.

#### 2.7.2. Plant Growth Parameters

At the end of drought stress treatment, the height and branch number of the *A. ordosica* plants were measured. The shoots and roots from each pot were separately harvested, and the roots were gently washed with tap water to remove the residual soil on their surface. Individual root samples were first suspended at an approximately 1 cm depth in deionized water in a plexiglass tray and then scanned using a scanner (EPSON V800, Seiko Epson Inc., NKS, Tokyo, Japan). The root morphological indexes were analyzed by the WinRHIZO image analysis system [57]. The roots were collected after scanning and a few root samples were randomly selected to analyze the DSE colonization (see below). After the physiological indicators were determined, the remaining root and shoot samples were dried in an oven at 70 °C for more than 48 h to measure the plant biomass.

#### 2.7.3. Plant Physiological Parameters

After the stress treatment, a spAD-502 chlorophyll content analyzer (Konica Minolta Sensing, Osaka, Japan) was used to determine the chlorophyll content of the *Artemisia ordosica* seedlings. The SPAD values were measured in random order from 9:00 to 11:00 a.m., and the chlorophyll content of each pot was calculated from the average value of the two plants [58].

The chemical composition and antioxidant enzyme activity of the shoots of the plant were determined, including the auxin content, SOD activity, and glutathione (GSH), MDA, soluble protein, and proline contents. The GSH content was determined using the DTNB method [59]. An acid ninhydrin colorimetry approach was applied to measure the proline content according to Bates et al. [60]. The root auxin (IAA) content was determined using the ELISA method [61].

### 2.8. Statistical Analysis

Analysis of variance (ANOVA) was performed with SPSS version 20.0 (SPSS Inc., Chicago, IL, USA). All data were tested for homogeneity of variances and normality using Levene’s test and the Shapiro–Wilk test. Data that did not fulfil the above assumptions were log-transformed. Then, *t*-test analysis was conducted for detecting the drought tolerance of each examined DSE strain compared with normal treatment, such as the biomass, SOD activity, and melanin content. Two-way ANOVA was used to analyze the effects of DSE inoculation, drought, and their interaction on the growth and physiological parameters of the *A. ordosica* plants. The differences between the means among the different treatments were compared using Tukey’s test at *p* < 0.05. The effects of the soil variables on the IFs of the DSE strains were examined by Pearson’s correlation analysis, and heatmaps were processed and formulated using R with the pheatmap package.

## 3. Results

### 3.1. DSE Colonization in Artemisia ordosica

**Figure 1:**
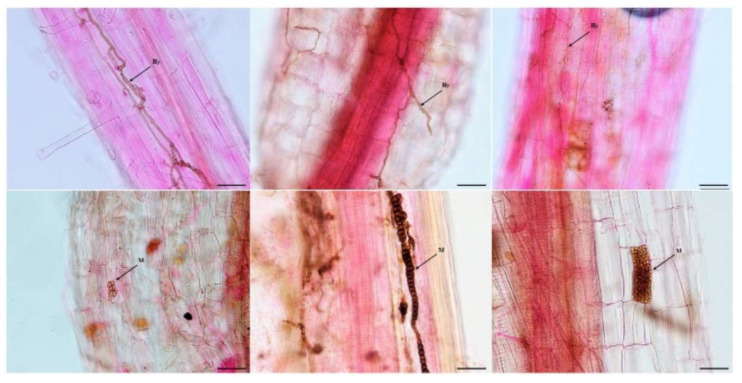
Dark septate endophytes associated with the roots of *Artemisia ordosica* growing in natural habitats. bars = 50 µm. Arrows indicate the following: Hy, DSE hyphae; M, DSE microsclerotia.

**Figure 2:**
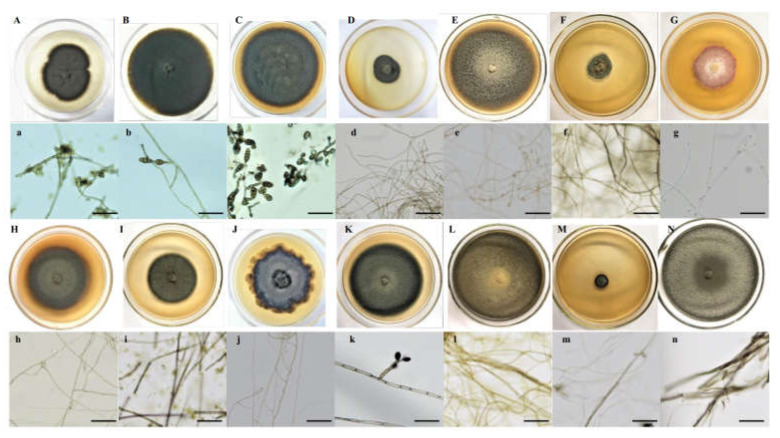
Colonies of dark septate endophytes isolated from the roots of *Artemisia ordosica* grown on potato dextrose agar (PDA) medium (**A**–**N**). (**a**–**n**), microscopic morphology of endophytic fungi (bars = 50 μm).

Melanized and septate hyphae and diverse microsclerotia were observed in the roots of *A. ordosica* growing naturally in an arid desert (Figure 1). For different sites, the IF of DSE in DK (55.0%) was significantly lower than that in ALS (66.7%) and SPT (73.3%) (*p* = 0.004). After 15 days of culture, morphological observation showed different types of DSE colonies that were 2.0–8.5 cm in diameter, round or oval, and mainly gray-black or brown in color (Figure 2). The surface of the colonies was mostly blank or felt-like and relatively flat, with a few surface ridges or concentric circles. Observation under a light microscope showed that the mycelia all had obvious septa that were mainly brown in color. Most of the DSE strains did not produce spores, while some produced inverted rod-shaped brown conidia and spherical conidia.

**Figure 3:**
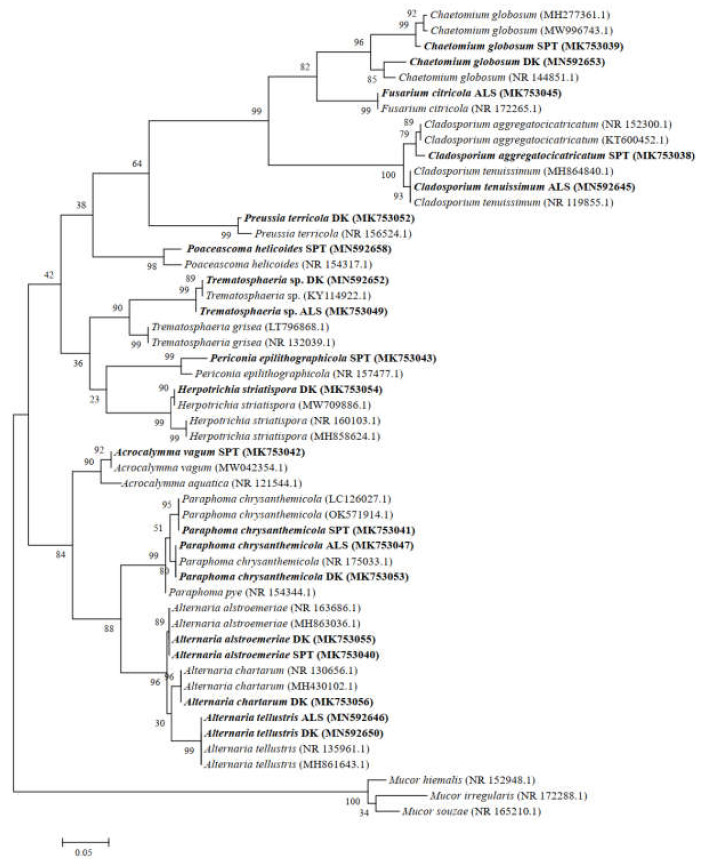
Maximum likelihood tree based on the ITS4-5.8S-ITS5 rDNA sequence analysis of DSEs isolated from roots of *Artemisia ordosica*. Sequences that were determined in the course of this study appear in bold.

**Figure 4:**
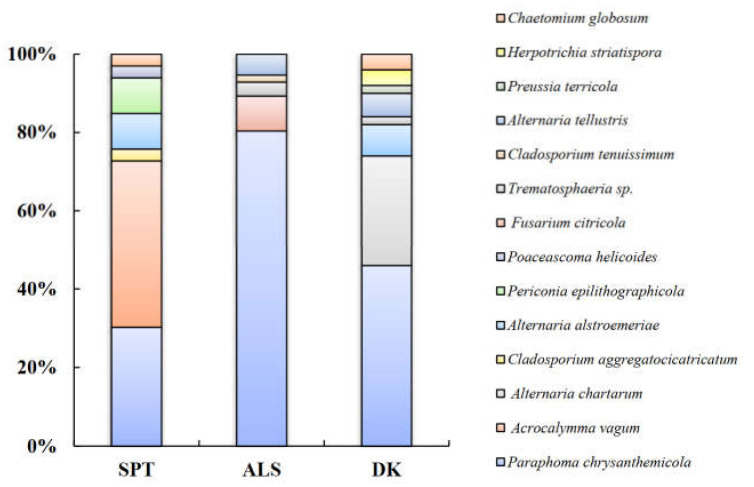
Column chart of the proportion of DSE species at three sample sites. Note: SPT, Shapotou. ALS, Alxa. DK, Dengkou.

**Figure 5:**
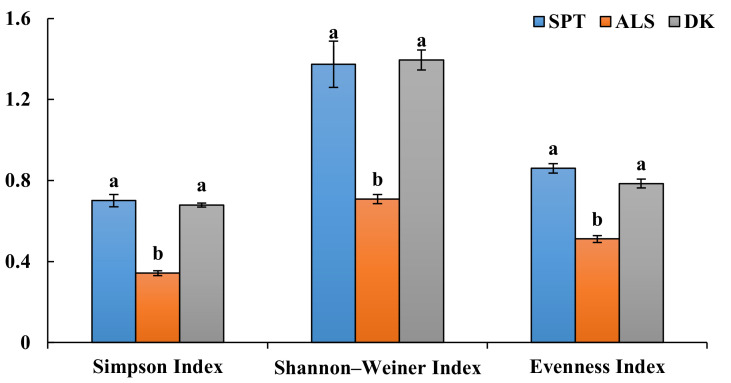
Assessment of the species diversity indices and colonization rate of DSE fungi at three sample sites. Note: SPT, Shapotou. ALS, Alxa. DK, Dengkou. Different letters above the error bars indicate a significant difference at *p* < 0.05.

According to the morphological identification and ITS sequence analysis, a total of 11 genera and 14 species of DSE fungi were isolated and identified from the roots of *A. oraceae* (Figure 3). These 11 genera were *Acrocalymma*, *Alternaria*, *Chaetomium*, *Cladosporium*, *Fusarium*, *Herpotrichia*, *Paraphoma*, *Periconia*, *Poaceascoma*, *Preussia*, and *Trematosphaeria*. The maximum values of the DSE species appeared in Dengkou, followed by Shapotou, while Alxa had the least species (Figure 4). The DSE diversity in SPT and DK was considerably higher than that of ASL (Figure 5). The highest values of the Simpson index and evenness indices were recorded at SPT site, while the Shannon–Weiner index was the highest at DK.

**Figure 6:**
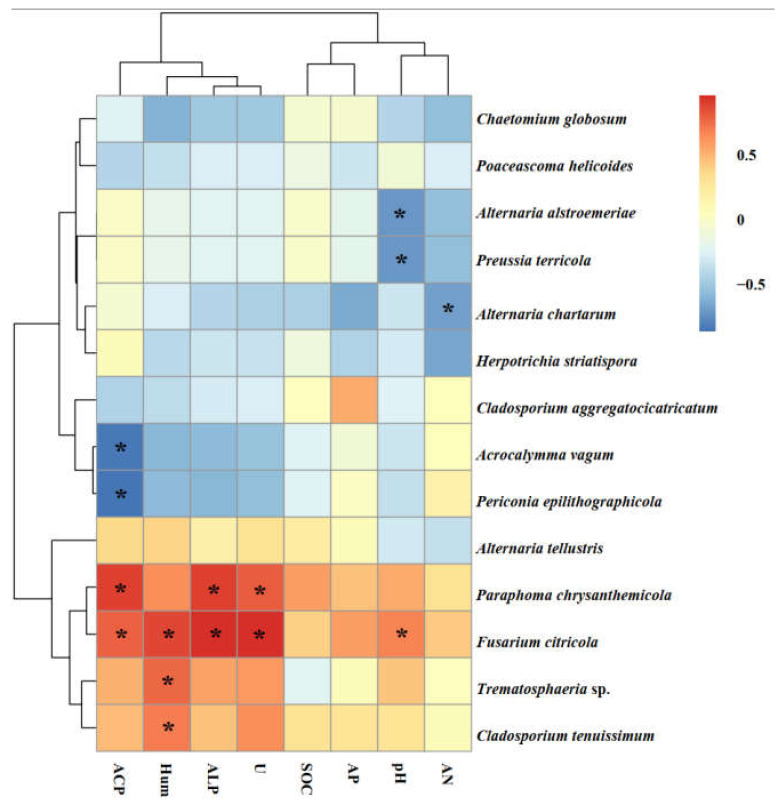
Heat maps of the correlation analysis between soil factors and the roots relative abundance of isolated endophytes. Note: ACP, soil acid phosphatase. Hum, soil moisture. ALP, soil alkaline phosphatase activities. U, soil urease. SOC, soil organic matter. AP, soil available phosphorus. AN, soil available nitrogen. The asterisks indicate significant correlations at *p* < 0.05.

The species *Paraphoma chrysanthemicola* was common to these three sites and was the dominant species in Alxa and Dengkou. *Alternaria alstroemeriae* was a species common to Shapotou and Dengkou (Figure 6). *Trematosphaeria* sp. and *Alternaria tellustris* were common to Alxa and Dengkou. Among all of the isolated fungi, *P. chrysanthemicola*, *Alternaria chartarum*, and *Acrocalymma vagum* were the dominant species, with isolation frequencies of 60.00%, 10.77%, and 10.77%, respectively. The correlation analysis results show that the IFs of *A. alstroemeriae* and *Preussia terricola* were significant negatively correlated to pH, while *Fusarium citricola* showed a positive relationship (Figure 6). The occurrences of *A. chartarum* were affected by AN. *Acrocalymma vagum* and *Periconia epilithographicola* were negatively affected by ACP. The Ifs of *P. chrysanthemicola* and *F. citricola* were closely corelated to ACP, ALP, and U. Moreover, *F. citricola*, *Trematosphaeria* sp., and *Chaetomium globosum* were positively affected by Hum.

### 3.2. In Vitro Drought Stress Tolerance of the DSE Stains

**Figure 7:**
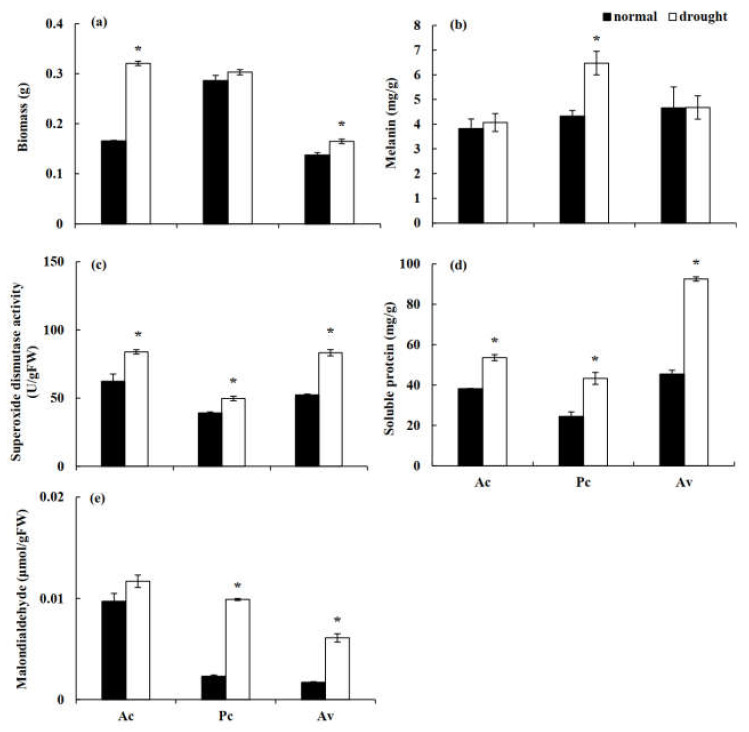
Biomass (**a**), melanin content (**b**), superoxide dismutase (SOD) activity (**c**), soluble protein (**d**), and malondialdehyde (**e**) content of three dark septate endophytes (DSEs) exposed to drought stress induced with polyethylene glycol (PEG) 6000. The asterisk above the error bars indicates significant difference between the normal and drought treatment of each DSE stain at *p* < 0.05. Note: Ac, *Alternaria chartarum*. Pc, *Paraphoma chrysanthemicola*. Av, *Acrocalymma vagum*.

The three studied DSE strains all grew well under stress conditions and showed a high drought tolerance capacity in vitro. Under drought stress, the biomass of the tested fungi showed an increasing trend, and the biomass of Ac and Av were significantly higher than that of the control (Figure 7a). The analysis of fungal melanin showed that the melanin content of Pc was significantly higher than that of the control under drought treatment, while those of Ac and Av were not significantly different from the unstressed treatment (Figure 7b). Under drought treatment, the activity of SOD and the soluble protein content of all DSE fungi were significantly higher than those of the control (Figure 7c,d). Compared to the control treatment, the MDA content of DSEs was significantly increased by drought stress, and the difference in Pc and Av was significant (Figure 7e).

### 3.3. Effect of DSEs on the Performance of Artemisia ordosica

**Figure 8:**
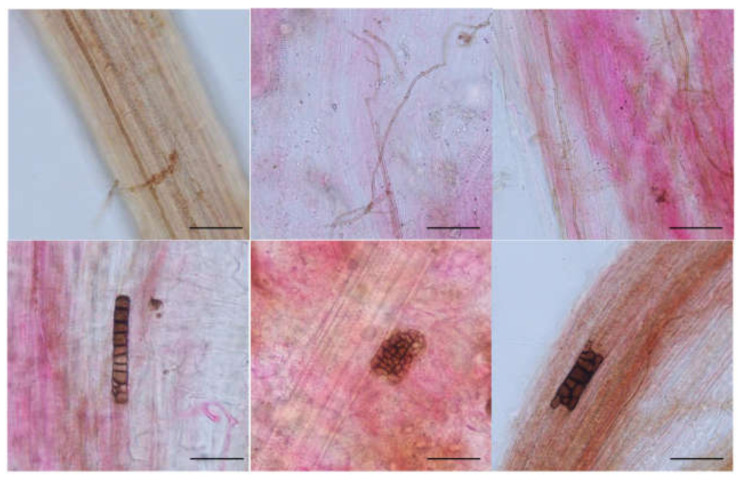
Colonization of DSE strains in the roots of inoculated *Artemisia ordosica* seedlings after harvest. Note: bars = 50 µm.

**Figure 9:**
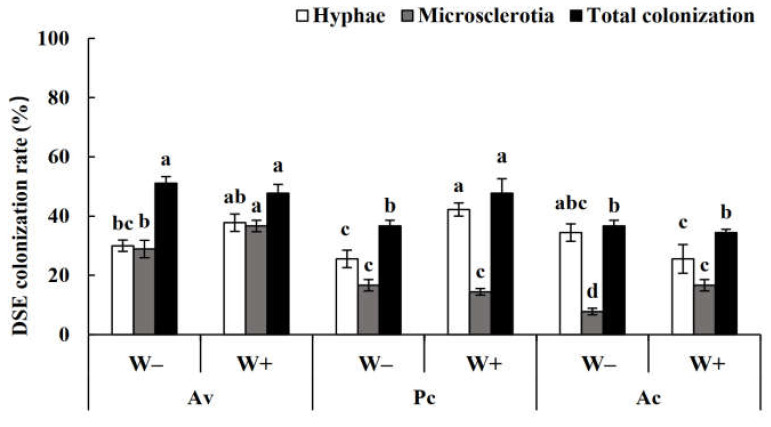
Dark septate endophyte (DSE) colonization rate in the roots of *Artemisia ordosica* inoculated with DSEs under drought stress. Note: W−, drought treatment. W+, well-watered treatment. Ac, *Alternaria chartarum*. Pc, *Paraphoma chrysanthemicola*. Av, *Acrocalymma vagum*. Different letters above the error bars indicate a significant difference at *p* < 0.05.

After harvest, no DSE colonization was found in the roots of the uninoculated plants, while dark septa or microsclerotia structures were observed in the roots of the inoculated *A. ordosica* plants (Figure 8). DSE fungal colonization was measured under different treatments. The colonization rate of the mycelia was 25–50%, while that of the microsclerotia was 2–40%, and the total colonization rate was 10–60% (Figure 9). Following inoculation, *A. vagum* showed stronger colonization than *P. chrysanthemicola* and *A. chartarum*.

#### 3.3.1. Vegetative Growth of *Artemisia ordosica*

**Figure 10:**
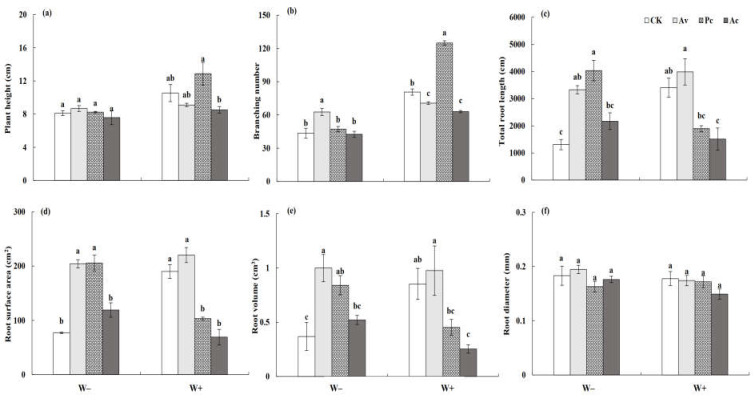
Effects of dark septate endophyte (DSE) on the shoot and root morphological traits of *Artemisia ordosica* seedlings under drought stress. Different letters above the error bars indicate a significant difference at *p* < 0.05. Note: (**a**), plant height, (**b**), branching number, (**c**) total root length, (**d**), root surface area, (**e**), root volume, (**f**), root diameter. W−, drought treatment. W+, well-watered treatment. CK, non-DSE inoculated control. Ac, Pc, and Av, plants inoculated with *Alternaria chartarum*, *Paraphoma chrysanthemicola*, and *Acrocalymma vagum*.

The seedlings of *A. ordosica* grew healthily during the experiment. Two-factor variance analysis showed that DSE inoculation, drought stress, and their interaction significantly affected the plant height, branch number, and root growth of *A. ordosica* (Table 1). The plant height and branch number were negatively affected by drought stress, but inoculation with DSE played a positive role in alleviating the adverse effects of drought stress on the growth of *A. ordosica* (Figure 10a,b). Although drought treatment reduced the plant height, the decrease in *A. ordosica* seedlings inoculated with Av and Ac was lower compared to that with the other treatments. Moreover, DSEs did not adversely affect the host height even under drought conditions. Under W− conditions, inoculation with DSEs increased the number of host branches, and Av exhibited a significant effect. Under the conditions of W+, Pc showed a significant positive effect on the host branching number, while Av and Ac had a significant negative effect. DSEs promoted the root growth of *A. ordosica*, especially under drought conditions (Figure 10c–f). The root length, root surface area, and root volume of *A. ordosica* inoculated with Av and Pc were significantly higher than those of the control plants. However, the root length and root surface area of *A. ordosica* inoculated with Pc and Ac were significantly reduced compared to the control under the W+ conditions, while the root of *A. ordosica* inoculated with Av was not significantly affected. DSE inoculation had no significant effect on the root diameter of *A. ordosica*

#### 3.3.2. Biomass Production of *Artemisia ordosica*

**Figure 11:**
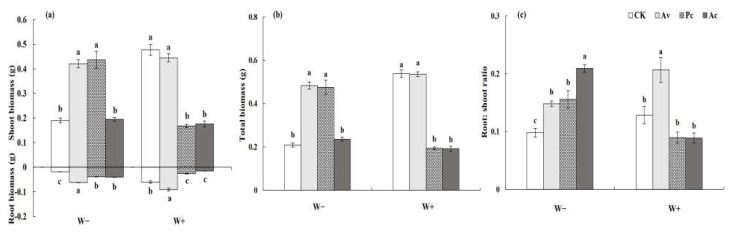
Effects of dark septate endophyte (DSE) on the biomass production and root–shoot ratio of *Artemisia ordosica* seedlings under drought stress. Different letters above the error bars indicate a significant difference at *p* < 0.05. Note: (**a**), shoot and root biomass, (**b**), total biomass, (**c**), root:shoot ratio. W−, drought treatment. W+, well-watered treatment. CK, non-DSE inoculated control. Ac, Pc, and Av, plants inoculated with *Alternaria chartarum*, *Paraphoma chrysanthemicola*, and *Acrocalymma vagum*.

DSE inoculation, drought, and their interaction significantly affected the biomass production and root–shoot ratio of *A. ordosica* (Table 1). Under drought conditions, the shoot, root, and total biomass of *A. ordosica* inoculated with Av were significantly higher than those of the control plants, showing an obvious growth promotion effect (Figure 11a,b). However, Av had no significant effect on the biomass accumulation of the host with a sufficient water supply. Nevertheless, the root–shoot ratio of the Av inoculated plants was significantly higher than that of the uninoculated plants due to their higher root biomass under W+ conditions (Figure 11c). Pc and Ac had beneficial effects on *A. ordosica* under drought treatment but detrimental effects under a sufficient water supply. Under W− conditions, the shoot, root, and total biomass of those plants inoculated with Pc were significantly higher than those of the control (Figure 11a,b). However, under W+ conditions, DSE inoculation showed a negative effect. Under W+ conditions, the biomass of the Ac plants was significantly lower than that of the control plants, but under drought conditions, this adverse effect disappeared, and the root–shoot ratio of the inoculated plants was significantly higher than that of the control plants (Figure 11c).

#### 3.3.3. Physiological Response Index of *Artemisia ordosica*

**Figure 12:**
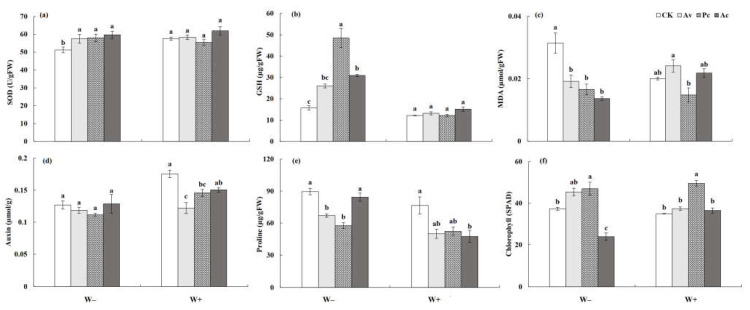
Effects of dark septate endophyte (DSE) on the shoot and root physiological traits of *Artemisia ordosica* seedlings under drought stress. Different letters above the error bars indicate a significant difference at *p* < 0.05. Note: (**a**), SOD, (**b**), GSH, (**c**), MDA, (**d**), auxin, (**e**) proline, (**f**), chlorophyll. W−, drought treatment. W+, well-watered treatment. CK, non-DSE inoculated control. Ac, Pc, and Av, plants inoculated with *Alternaria chartarum*, *Paraphoma chrysanthemicola*, and *Acrocalymma vagum*.

In general, DSEs increased the activity of antioxidant enzymes in *A. ordosica* under drought conditions (Table 2). Under W− treatment, the SOD and GSH activities of *A. ordosica* inoculated with DSEs were all significantly higher than those of the control plants, while no significant effect was observed under W+ conditions (Figure 12a,b). Meanwhile, DSE fungi played an active role in reducing the MDA content of the host plant under drought stress (Figure 12c). The auxin content of *A. ordosica* decreased under drought stress. Although DSEs showed no significant effect on the auxin content of *A. ordosica* under drought conditions, the auxin content decreased to a lesser degree in the inoculated plants than the control plants, while the auxin content of Pc-inoculated *A. ordosica* exhibited an increasing trend (Figure 12d). Under W+ conditions, the proline content of *A. ordosica* inoculated with Ac was significantly lower than that of the control, but there was no significant difference between them under drought conditions. The results show that Ac inoculation had a stronger promoting effect on the proline content of the host plant under drought conditions (Figure 12e). Pc significantly promoted the chlorophyll content in *A. ordosica* under all treatments (Figure 12f).

## 4. Discussion

### 4.1. Colonization and Species Diversity of DSEs in Artemisia ordosica

In this study, 14 DSE strains belonging to 11 genera were isolated from the roots of *A. ordosica* by morphological and molecular identification. All isolated strains produced a dark septate mycelium and other structures in the culture or root system, suggesting that they could be considered DSEs. All of these strains belong to Ascomycota, and most of them belong to Pleosporales. This is consistent with previous reports on desert DSEs in that Pleosporales are frequently isolated in arid environments [40,62,63]. The species composition of the DSEs was significantly different among the sampling sites, with obvious spatial heterogeneity. *Paraphoma chrysanthemicola* was a common species in these three sites and had the highest isolation rate. Meanwhile, the DSE fungi isolated in this study are widely distributed in various ecosystems. Among them, *P. chrysanthemicola*, *H. striatispora*, *A. vagum*, and *A. alstroemeriae* have been isolated from several desert plants, such as *Hedysarum scoparium*, *Reaumuria soongorica*, *Sympegma regelii*, *Ephedra przewalskii*, *Nitraria sphaerocarpa*, *Salsola passerine*, *Ammopiptanthus mongolicus*, and *Glycyrrhiza uralensis* [63,64,65]. The plants are greatly affected by the soil environment, where most of their symbiotic fungi come from [66,67]. Therefore, the relatively consistent environmental conditions in desert habitats may explain the similarity of plant symbiotic DSE fungi to a certain extent. In addition, *P. chrysanthemicola* and *A. vagum* have also been found in *Nicotiana tabacum*, *Pinus tabulaeformis*, *Oryza sativa*, and other plant root tissues [68,69]. This indicates that DSE fungi show no significant host specificity and strong ecological adaptability.

The soil microenvironment can affect the plant growth and microorganism distribution and then alter the stability of a desert ecosystem [70,71,72]. Among them, the soil pH value is an important factor affecting the distribution and diversity of symbiotic fungi, as it plays a key role in the soil nutrient availability [73,74]. In this study, the IFs of most of the DSE fungi showed a negative correlation with the pH value, indicating that these fungi have a preference for acidic soil. This has also been reported in other studies on desert plants [66]. Meanwhile, a large number of studies have shown that soil nutrients may significantly impact the soil fungal community [75,76]. In this study, the IF levels of *A. chartarum* and *H. striatispora* were negatively correlated with the soil total nitrogen content, consistent with previous studies [77,78]. This might be related to the utilization of nitrogen source nutrients by DSEs. When plant roots are deficient in inorganic nitrogen, the increase in DSE colonization of plant roots can mineralize rhizosphere peptides, amino acids, and proteins, thus improving the acquisition of soil inorganic nitrogen by plants [17]. Soil enzymes, the main exudates of soil microorganisms and plant roots, are important indicators that reflect the intensity of soil biochemical processes and are used to evaluate soil fertility [79,80]. For instance, soil urease is an important catalyst for the decomposition and transformation of soil urea, and its activity reflects the nitrogen utilization rate of plants and microorganisms to a certain extent [81,82]. In this study, *P. chrysanthemicola* and *F. citricola* were positively affected by urease activity, suggesting that these two DSE fungi may participate in the transformation of soil nitrogen and thus prevent or attenuate its accumulation. In addition, the IFs of DSE fungi are closely related to soil moisture, and most showed a negative response. This may indicate that the high drought tolerance capacity of DSE strains and the water supply of the natural habitat is still unfavorable for DSE growth [38].

### 4.2. DSE Growth under Drought Stress

Previous studies have shown that DSE fungi are widely distributed in arid and semiarid environments and can improve the tolerance of host plants to drought stress [18,26,27,63,83,84,85]. In this study, all three of the DSE strains isolated from *A. ordosica* grew well under drought stress, and the biomass accumulation of Ac and Av was significantly higher than that of the control. We also found similar phenomena in a study of other desert fungi [27,63,83], suggesting that endophytic fungi in water-stressed habitats generally confer certain advantages to hosts in terms of drought resistance.

The antioxidant system comprises an important strategy for plants and microorganisms to cope with oxidative damage [86,87,88]. SOD is considered to be an important antioxidant enzyme to eliminate the reactive oxygen species (ROS) produced under oxidative damage [87,89]. In this study, the SOD activity of the three DSE fungi significantly increased under drought stress, suggesting that DSE fungi may accumulate SOD to remove ROS, thereby greatly reducing oxidative damage to DSE fungi. Li et al. [90] also found that DSE inoculation can increase the SOD activity of *Isatis indigotica* under drought stress conditions. Under drought stress, mycelium cells may lose water, result in decreased cell turgor, which affects substance metabolism and even inhibits mycelium growth [91]. As an osmotic regulator, soluble protein can directly promote osmotic regulation and improve the drought tolerance of cells [54]. In this study, the soluble protein content of these three fungi was significantly higher than that of the control, indicating that soluble protein is directly involved in osmotic regulation and enhances the tolerance to dehydration under water deficit. In addition, melanin, as the main component of DSE fungal cell walls, is believed to improve the structural rigidity of the cell wall and reduce the oxidative damage associated with stress environments, thus enhancing fungal stress tolerance [56,64,92]. In our study, the melanin content of Av and Ac did not increase under drought treatment, indicating that this treatment does not induce melanin biosynthesis in them. However, the content of melanin in Pc was significantly higher than that in the control, suggesting that fungal melanin may play a role in specific fungi. It is possible that melanin plays a role in the tolerance to other types of abiotic stress depending on the fungi. Gaber et al. [93] also found that melanin was not necessary for salt stress resistance of DSE strains. In conclusion, Av and Ac showed higher resistance to drought stress in vitro, which may be related to the regulation of the antioxidant enzyme system. The growth of Pc was not adversely affected under drought stress, which may be related to having a higher melanin content or a result of other mechanisms.

### 4.3. Effect of DSEs on the Performance of Artemisia ordosica

Endophytic fungi can form complex symbiotic relationships with plants and are widely distributed in many ecosystems [94,95]. Studies have shown that endophytic fungi can colonize the roots of various plants under drought stress and improve the tolerance of host plants to drought through the involvement of complex biological pathways [17,18,20,21,22]. In this study, DSE fungi were found to have colonized *A. ordosica* root tissues under all inoculated treatments, indicating that these desert fungi are still effective root colonizers of *A. ordosica* under water deficit conditions. Our results also showed that the mycelium was the main colonization structure of DSEs in *A. ordosica* roots under all the treatments. The mycelia crossing the plant cell walls and penetrating into plant cells and intercellular spaces could facilitate water transport and nutrient exchange between plants and fungi. This is an important ecological strategy for fungi in resisting stress environments [17,96]. In addition, the roots of *A. ordosica* were colonized by microsclerotia under stress. In previous reports, microsclerotia were considered to be a propagule or hypopus. Growth of these microsclerotia, with their spore-like structure, may be another important strategy employed by DSE fungi in resisting drought stress [18,32,96].

The effects of DSE inoculation on *A. ordosica* growth under drought stress varied but were generally beneficial. Additionally, the responses resulting from interactions between the DSEs and plants were dependent on the DSE species involved. Av and Pc had obvious effects on the growth and drought resistance of *A. ordosica*. Under drought treatment, the biomass accumulation and vegetative growth of *A. ordosica* inoculated with DSEs were significantly higher than those of the control. This growth-promoting effect may be related to the enhanced root growth of the host. In this study, DSE inoculation increased the root length and root surface area of *A. ordosica* and reduced the adverse effects of drought. In related studies on other plants, Wu et al. [97] found that DSE stimulated the root development of an endangered medicinal plant. Liu et al. [98] found that DSE could help *Ormosia hosiei* seedlings adapt to a drought stress environment by altering the root morphology and reducing ultrastructural damage. A well-developed root system and a higher branch number are extremely important for the growth of desert plants, so they may have resulted from the long-term coevolution of DSEs and desert plants. Deep and extensive root development of host plants can promote the absorption of water and nutrients and ultimately affect plant biomass production by improving subsurface and aboveground morphological indicators to improve the adaptability of host plants to drought stress [22]. Positive effects of Av and Pc on *A. ordosica* were also found to be related to the regulation of the antioxidant enzyme system. Under drought stress, SOD activity and GSH contents were significantly increased in *A. ordosica* seedlings inoculated with DSE. The presence of these two substances is essential for reactive oxygen species removal in plants [38,57,99]. Moreover, MDA is an important biomarker for assessing the degree of oxidative damage in plants [100,101]. In this study, the MDA content of *A. ordosica* was significantly reduced after inoculation with Av and Pc under drought stress, suggesting that DSEs alleviate the adverse effects of drought stress on plants. In general, Av and Pc mainly enhance the adaption of host plants to adverse conditions by promoting plant growth as well as the antioxidant enzyme activity and root development of hosts under drought stress.

Although Ac exhibited a high separation rate in *A. ordosica* roots, it did not show an obvious growth-promoting effect in the inoculation test that was expected. After harvest, the biomass of *A. ordosica* inoculated with Ac under drought treatment was not significantly different to that of the control, but the root–shoot ratio of the host significantly increased. The analysis of physiological indexes shows that Ac increased the SOD activity and GSH content in *A. ordosica* under drought conditions. At the same time, the increase in the proline content of Ac-inoculated *A. ordosica* was significantly higher than that of the control under drought conditions. Previous studies have also found that inoculation of fungi can increase the accumulation of osmotic adjustment substances and reduce the osmotic potential of host cells, so as to resist dehydration injury caused by stress [102,103,104]. This is in accordance with a recent study by He et al. [65], who found that *A. vagum* can enhance the drought tolerance of the host plant *Astragalus mongholicus*. In natural habitats, the higher isolation rate of Ac may be related to the enhancement of stress resistance and the guarantee of normal growth of *A. ordosica* under drought conditions. It may also be that the effect of Ac on the host was not enough to affect the accumulation of biomass, but if the assessment is carried out after a longer period, the DSEs will eventually promote plant growth.

Plant growth is usually inseparable from the participation and regulation of plant hormones. The ability of endophytic fungi to produce hormones by themselves or by promoting plant production is beneficial for host resistance to adverse effects under stress conditions [12,61,105,106]. As the main endogenous hormone in plants, IAA plays an important role in regulating plant responses to external stress. Qiang et al. [12] found that inoculation with *A. alternata* could increase the auxin content, improve the growth, and enhance the drought stress tolerance of wheat. In addition, studies on other microorganisms also found that AM fungi could increase the auxin content of *L. barbarum*, promote plant osmotic regulation, and increase plant tolerance to salt stress [107]. Based on its growth-promoting effect, we expected DSEs to promote auxin accumulation in *A. ordosica* plants. In this study, although inoculation with DSEs had no significantly beneficial effect in increasing the host auxin content, the extent of decrease in auxin content was lower in inoculated plants than of control plants under drought conditions. Moreover, under drought, the auxin content of Pc-inoculated *A. ordosica* showed an increasing trend. These results indicate that DSEs have a certain effect on the auxin regulation of *A. ordosica* under drought stress. In addition, the chlorophyll content was significantly higher in Pc-inoculated *A. ordosica* plants than the control under all treatments. Chlorophyll is an important pigment for photosynthesis and plays an important role in the process of light absorption [108]. Under drought stress, DSEs may affect plant growth by increasing the host’s chlorophyll content and thereby increasing the photosynthetic rate. Other studies have also found that endophytic fungal infection can increase the photosynthetic pigment content of host plants to improve plant growth under stress [109,110].

Afforestation is still the fundamental method for vegetation restoration in arid regions in China. To ensure this is effective, it is important to improve the growth and survival ability of *A. ordosica* seedlings under drought stress. In this study, we found that three dominant DSE fungi isolated from deserts could exert beneficial effects by promoting host growth or enhancing their drought tolerance, which might be one of the reasons for their high isolation rate in natural habitats. Based on the results of this study, we speculate that DSE–*A. ordosica* symbionts could play an important role in promoting vegetation growth and ecological restoration in arid regions.

## 5. Conclusions

This study investigated the species diversity and promoting potential of DSEs associated with a dominant subshrub growing naturally in the desert of northwest China. We found that DSEs are universally observed in *A. ordosica* roots, and a total of 14 DSE species grouped into 11 genera were identified. Three dominant DSE species cultured in vitro showed good drought resistance, and they also promoted the growth of *A. ordosica* under drought conditions. In general, *A. vagum* and *P. chrysanthemicola* had better promotion effects on the biomass accumulation, root development, and antioxidant enzyme activity of *A. ordosica*, leading to better growth and drought resistance. Considering that *A. ordosica* plays vital roles in preventing wind erosion and fixing sand in deserts, our findings are crucial for understanding how plants combat drought and to provide evidence for the application of DSEs in vegetation restoration in arid ecosystems.

## Figures and Tables

**Table 1:** Analysis of variance for the effects of drought stress and dark septate endophyte (DSE) inoculation on the shoot and root morphological traits and biomass production of *Artemisia ordosica* seedlings.

	Plant Height		Branching Number		Total Root Length		Root Surface Area		Root Volume		Root Diameter		Shoot Biomass		Root Biomass		Total Biomass		Root:Shoot Ratio	
	*F*	*p*	*F*	*p*	*F*	*p*	*F*	*p*	*F*	*p*	*F*	*p*	*F*	*p*	*F*	*p*	*F*	*p*	*F*	*p*
drought	17.57	**0.001**	365.16	**< 0.001**	0.01	0.966	0.53	0.479	0.32	0.581	1.99	0.177	0.22	0.646	18.28	0.001	1.53	0.234	7.85	**0.013**
DSE	4.34	**0.020**	56.34	**< 0.001**	11.79	**< 0.001**	38.39	**< 0.001**	8.15	**0.002**	1.76	0.195	65.80	**<0.001**	127.37	< 0.001	111.41	**< 0.001**	11.08	**< 0.001**
drought × DSE	3.51	**0.040**	65.86	**<0.001**	15.74	**<0.001**	34.11	**<0.001**	4.96	**0.013**	1.07	0.391	82.25	**< 0.001**	67.72	< 0.001	119.85	**< 0.001**	23.19	**< 0.001**

Note: Significant *p*-values are in bold.

**Table 2:** Analysis of variance for the effects of drought stress and dark septate endophyte (DSE) inoculation on the physiological traits of *Artemisia ordosica* seedlings.

	SOD		GSH		MDA		Auxin		Proline		Chlorophyll	
	*F*	*p*	*F*	*p*	*F*	*p*	*F*	*p*	*F*	*p*	*F*	*p*
drought	3.08	**0.098**	193.48	**<0.001**	0.01	0.997	27.19	**<0.001**	32.16	**<0.001**	1.18	0.293
DSE	7.50	**0.002**	30.96	**<0.001**	10.99	**<0.001**	6.64	**0.004**	15.35	**<0.001**	49.69	**<0.001**
drought × DSE	3.48	**0.041**	31.55	**<0.001**	10.51	**<0.001**	3.39	**0.044**	4.42	**0.019**	16.12	**<0.001**

Note: SOD, superoxide dismutase activity. GSH, glutathione content. MDA, malondialdehyde content. Significant *p*-values are in bold.

## Data Availability

The data collected and analyzed throughout the present research are available upon request. Fungal sequences were deposited in NCBI GenBank and the accession numbers were given in the text.
